# Caregiver burden and its association with depression, anxiety, and quality of life among caregivers of individuals with severe persistent mental illness in Saudi Arabia: A cross-sectional study

**DOI:** 10.1371/journal.pone.0355872

**Published:** 2026-08-13

**Authors:** Fatimah Albrekkan, Shuliweeh Alenezi, Ahmad Ghesheyan, Mohammed S. Almousa, Mohammed A. Alabduljabbar, Ibraheem M. Alkanhal, Musaab A. Alzuhair, Ibrahim E. Alahmadi

**Affiliations:** 1 Department of Psychiatry, College of Medicine, King Saud University, Riyadh, Saudi Arabia; 2 College of Medicine, King Saud University, Riyadh, Saudi Arabia; University of Sao Paulo, BRAZIL

## Abstract

Caregivers of patients with Severe Persistent Mental Illness (SPMI) face tremendous challenges. They often experience elevated levels of burden, depression, and anxiety. These factors negatively impact their quality of life (QoL). However, few studies have examined caregiver burden and its psychological effects in Saudi Arabia. This study aims to assess caregiver burden in relation to socio-demographic characteristics and its correlation with depression, anxiety, and QoL. The study tool consisted of validated questionnaire, including the Patient Health Questionnaire-9 (PHQ-9), Generalized Anxiety Disorder-7 (GAD-7), abridged Arabic version of the Zarit Burden Interview (ZBI), and the World Health Organization Quality of Life-Brief Version (WHOQOL-BREF). A cross-sectional convenience sample of 120 participants was recruited from the Psychiatry Outpatient Clinics at King Saud University Medical City in Riyadh, Saudi Arabia. Our results showed a significant positive correlation between caregiver burden and depression and anxiety scores (p < 0.001). Moreover, significantly higher burden levels were found among female caregivers and those residing with patients, p = 0.04 and p = 0.002, respectively. Additionally, the severity of mental illness was also associated with an increased burden level (p = 0.001). Notably, spouses and children reported significantly higher burden levels (p = 0.036). In multivariable analyses, caregiver anxiety and perceived severity of the patient’s mental illness were independently associated with higher caregiver burden, while depressive and anxiety symptoms were independently associated with poorer quality of life.Furthermore, a significant negative correlation was found between caregiver burden and the physical and psychological QoL domains (p < 0.001 and p = 0.013, respectively). Collectively, our findings highlight the mental health challenges faced by caregivers of individuals with SPMI in Saudi Arabia, which negatively impact their QoL. Consequently, these findings substantiate the urgent need for targeted support interventions, particularly those addressing caregiver anxiety, depression, and the challenges associated with severe mental illness, especially for caregivers who live with or care for individuals with SPMI.

## 1. Introduction

Mental illness is a health condition that can cause changes in behavior, thinking, emotions, or a combination of these [1]. The term “Severe and Persistent Mental Illness" (SPMI) is commonly used to describe a group of mental disorders that typically emerge in early adulthood and significantly affect family relationships, education, work productivity, and social functioning throughout a person’s life [2]. However, there is no universally accepted definition of Severe and Persistent Mental Illness (SPMI) as there is significant variation in how its core dimensions—diagnosis, disability, and duration—are defined and operationalized. This inconsistency stems from differing contexts and purposes among researchers, policymakers, and clinicians, making it challenging to establish a standardized definition [3]. These illnesses include, but are not limited to, schizophrenia, bipolar disorder, major depressive disorder, personality disorders, persistent depressive disorder, obsessive-compulsive disorder, autism spectrum disorder, and attention-deficit hyperactivity disorder [3].

Mental illness is prevalent worldwide. In 2021, one in seven individuals, or 1.1 billion people, had a mental illness, with anxiety and depressive disorders being the most prevalent [4]. The Saudi National Mental Health Survey (SNMHS), part of the World Health Organization (WHO) World Mental Health (WMH) Surveys initiative, was one of the first national studies to establish the prevalence of mental illness in Saudi Arabia. A total of 4004 interviews were completed, of which 1981 respondents were included in the study. The study found that over 20.2% of respondents had some form of mental illness, with anxiety disorders being the most prevalent (12.3%). Mood disorders were present in 6.8% of respondents. Regarding individual disorders, social phobia was the most common, followed by major depressive disorder and separation anxiety disorder (4.2%, 3.8%, and 3.7%, respectively) [5].

The phrase *caregiver burden* refers to the complex stress experienced by individuals providing ongoing care to family members or loved ones [6]. Quality of Life (QoL) is the perception of one’s position in life in relation to goals, expectations, standards, and concerns within one’s cultural and value systems [7]. Several studies have shown that caregivers are at a higher risk of developing mental illnesses than the general population [8,9]. Caregivers are also more likely to experience psychological, social, and physical hardship [10]. A Brazilian study reinforced this perspective and found that caregivers have a high prevalence of depressive disorders [11]. Another study found that caregivers face profound psychological stress in addition to increased depression and anxiety, with elevated levels of caregiver burden [12]. Several socio-demographic factors, such as living with the patient [11] and having a low monthly income [13], were associated with increased burden levels.

While systematic reviews have highlighted the multidimensional impact of caregiving, including increased burden, emotional distress, and reduced well-being among family members of individuals with severe mental illness [10,12], evidence from Saudi Arabia remains limited. Existing local research has primarily focused on specific aspects of the caregiving experience, such as QoL among caregivers of individuals with mental illness [14]. Consequently, a more comprehensive understanding of caregiver burden and its relationship with depression, anxiety, and QoL among caregivers of individuals with SPMI in Saudi Arabia remains scarce. Given the cultural and social factors that may influence caregiving experiences in Saudi Arabia, further research is needed to better understand these interrelated outcomes. Therefore, this study aimed to examine caregiver burden and its association with depression, anxiety, and QoL among caregivers of individuals with SPMI in Saudi Arabia. By simultaneously examining caregiver burden, depression, anxiety, and QoL among caregivers of individuals with SPMI in Saudi Arabia, this study addresses an important gap in the regional literature and provides evidence to inform culturally appropriate caregiver support services and interventions.

## 2. Materials and methods

### 2.1 Design and ethical aspects

This is a cross-sectional study that was conducted at one of the tertiary hospital, King Saud University Medical City (KSUMC) psychiatry outpatient clinic in Riyadh, Saudi Arabia, from February 2024 to April 2024. For the purpose of this study, a caregiver was defined as an adult family member or significant other who provided unpaid physical, emotional, supervisory, or practical support to an individual with SPMI and who was involved in the patient’s care on a regular basis. Eligible participants were required to be aged 18 years or older and able to read and understand Arabic. Diagnosis include schizophrenia, bipolar disorder, major depressive disorder, personality disorders, persistent depressive disorder, and obsessive-compulsive disorder. Additionally, we included caregivers of patients with neurodevelopmental disorders such as autism and attention-deficit hyperactivity disorder (ADHD) in Saudi Arabia. These disorders were grouped under the broader category of SPMI because they are chronic conditions associated with substantial functional impairment, long-term care needs, and significant caregiving demands. To explore potential differences related to diagnosis, additional analyses were conducted according to diagnostic category and are reported in the Results section. Our exclusion criteria were caregivers of patients with neurocognitive disorders such as Alzheimer’s disease, which has been extensively studied in the literature.

The nature and purpose of the study, the principal investigator’s contact information, and an explanation of the confidentiality and data anonymity policy were provided. Before participating in the survey, the participants provided informed consent. After providing consent, the participants accessed the study survey, which took an average of 15–20 minutes to complete. The study was conducted in accordance with the Declaration of Helsinki, and approved by the Institutional Review Board of the College of Medicine, King Saud University (Research Project No. E-24–8487, dated 31/01/2024). Responses were collected anonymously, and no personally identifiable information was obtained. Data were stored securely and accessed only by the research team.

### 2.2. Sample

We conducted a pilot study to estimate our sample size using a single-mean formula by selecting the Arabic version of the Zarit Burden Interview scale with the highest available standard deviation. The standard deviation was 7.37. For the precision of the estimate (d), we chose a one-difference score for the total ZBI-A score. Zα/2 was 1.96 for a 95% confidence level. This led us to calculate the sample size as follows:

(1.96)^2^×(7.37)^2^/(1)^2^=3.841×54.32/1=208.66≈209 𝑝𝑎𝑟𝑡𝑖𝑐𝑖𝑝𝑎𝑛𝑡𝑠

We estimated that the non-response rate would be 20%; therefore, we added this estimate to the sample size: 209 + 41.8 ≈ 251. Considering that the standard deviations for the remaining scales were lower than those of the ZBI-A, the required sample size for these instruments would be less than 209 participants. However, we encountered several significant challenges that impacted our ability to meet the initially calculated sample size of 251 participants, resulting in a sample size of 120 participants. These difficulties arise primarily from niche population challenges, limited cooperation from relevant organizations, and time constraints. Moreover, we conducted a convenience sampling technique in which we interviewed every attending caregiver from the outpatient clinics at KSUMC.

### 2.3. Procedure

Caregivers who were eligible and attended the psychiatric outpatient clinic during the study period were approached consecutively by members of the research team, who explained the study’s objectives. Those who met the eligibility criteria and agreed to participate were invited to complete a survey. This survey could be done either electronically via a secure link or in person during their clinic visit. Participation was entirely voluntary, and no incentives were offered. Recruitment continued throughout the study period until no additional eligible caregivers were available to enroll.

The primary outcome variables (effects) we evaluated are as follows:

- Caregiver burden includes the time spent on oneself, role strain, anger, negative relationships, emotional strain, health-related strain, lack of privacy, social life, loss of control, decision uncertainty, perceived shortcomings, and self-efficacy.
- Caregivers’ QoL scores encompassed four domains: physical health, psychological health, social relationships, and environment.
- Depression scores.
- Anxiety scores.

The main assessed exposure variables (causes and risk factors) were as follows:

- Caregivers’ characteristics included sex, age, city of residence, marital status, education level, living with a person with mental illness in the same household, and monthly income.
- Characteristics of people with mental illness: sex, age group, relationship with caregivers, type of mental disorder, and type and quality of support

### 2.4. Measures/instruments

We conducted a survey across five sections. The first section consisted of socio-demographic questions. In the second section, we used the ZBI-A, a widely used tool comprising 12 items to assess the level of burden in caregivers of patients with different morbidities [6]. Items were rated on a 5-point Likert scale ranging from 0 (never) to 4 (almost always), with a higher score representing a higher sense of burden. The scale scores ranged from 0 to 48. It was initially written in English and translated into Arabic by Bachner et al. and was found to be valid and reliable [15].

The World Health Organization Quality of Life Questionnaire (WHOQOL-BREF), an abbreviated and validated version of the WHOQOL-100 assessment tool, is used in the third section. The WHOQOL-BREF was developed to evaluate four QoL domains: physical health (seven items), psychological health (six items), social relationships (three items), and environment (eight items). It consists of 26 items, with 24 items divided into four domains and two additional general items on overall QoL and general health. Each item is rated on a 5-point Likert scale, where a higher score indicates better QoL within a particular domain [16,17]. The Arabic version of the WHOQOL-BREF has demonstrated high reliability and validity indices [14].

In the fourth section, we used the Generalized Anxiety Disorder-7 (GAD-7) scale, a widely used tool for assessing the severity of anxiety symptoms. It consists of seven items scored from 0 to 3, with total scores ranging from 0 to 21. Higher scores indicated more severe anxiety. The GAD-7 has been demonstrated to be reliable and valid in various populations [18]. The Arabic version of the GAD-7 demonstrated reasonable reliability [19].

In the fifth section, we utilized the Patient Health Questionnaire-9 (PHQ-9), a validated tool used in various populations, and conducted studies in different languages to assess depression severity. Items were scored from 0 to 3, ranging from 0 to 27, with a cutoff value of 10 [20]. The Arabic version has good internal consistency with a Cronbach’s alpha of 0.857 [19].

### 2.5. Statistical analysis

In the data analysis section, we employed a range of statistical methods to examine the collected data using SPSS version 21. The data distribution and central trends are summarized. We applied Student’s t-test for independent samples to compare the differences between the two independent groups. For comparisons among more than two groups, we used Analysis of Variance (ANOVA), which allowed us to assess the statistical significance of differences across multiple categories, such as income levels or different types of relationships with the patient (e.g., spouses, children, or uncles).

Prior to conducting parametric analyses, the assumptions of normality and homogeneity of variance were evaluated. Normality of continuous variables was assessed using the Kolmogorov–Smirnov test and visual inspection of histograms. Homogeneity of variances was assessed using Levene’s test for independent-samples t-tests and ANOVA. For multivariable linear regression, multicollinearity was evaluated using tolerance statistics and variance inflation factor (VIF) values. Residual plots and observed-versus-predicted distributions were examined to assess model fit.

Multivariable linear regression analyses were performed to identify factors independently associated with caregiver burden and caregivers’ quality of life. Separate models were constructed using the Zarit Burden Interview (ZBI) score and the overall Quality of Life (QoL) score as dependent variables. Candidate predictor variables were selected based on their clinical relevance, evidence from the literature, and findings from the preliminary bivariate analyses. An exploratory iterative modelling approach was used to develop parsimonious final models while evaluating multicollinearity, model fit, and clinical interpretability. The final caregiver burden and quality-of-life models included six and eight predictors, respectively, representing approximately 20 and 15 observations per predictor, indicating an adequate participant-to-predictor ratio for the multivariable analyses. The associations between the independent predictor variables and the outcomes were expressed as un-standardized beta coefficients with corresponding 95% confidence intervals. Variables demonstrating substantial conceptual or statistical redundancy were excluded where appropriate to avoid collinearity inflation. A two-sided p-value < 0.05 was considered statistically significant.

## 3. Results

### Caregiver burden by sociodemographic characteristics

Descriptive statistics for the Zarit Burden Interview (ZBI) by socio-demographic characteristics of caregivers are shown in Table 1. When caregiver burden was analyzed by sex, females had a higher mean total ZBI score (M = 15.74, SD = 9.76). Males had a lower mean score (M = 12.05, SD = 9.72). Divorced caregivers had the highest mean total ZBI score (M = 18.78, SD = 10.50).

**Table 1:** Descriptive statistics of Zarit Burden Interview scores (ZBI) by caregiver socio-demographic characteristics.

		N	Mean	Standard Deviation
Gender	Female	57	15.74	9.76
	Male	63	12.05	9.72
Marital status	divorced	9	18.78	10.50
	Married	55	14.45	10.40
	Single	53	12.15	8.95
	widow	3	16.00	12.17
Educational level	High School	19	9.42	6.13
	Higher education	99	14.81	10.25
	Middle School	2	5.50	4.95
Monthly income in Saudi Riyals (SR)	Less than 5000	33	13.39	8.99
	5000–10000	19	13.37	8.89
	10000–20000	36	12.89	9.76
	More than 20000	32	15.50	11.53
Living with the patient	No	37	9.73	7.81
	Yes	83	15.61	10.19

Regarding educational level, caregivers with a middle school education reported the lowest burden (M = 5.50, SD = 4.95). However, it should be noted that this group had a small sample size (n = 2), which may limit the reliability of this result. Caregivers with a high school education had a mean ZBI total score of 9.42 (SD = 6.13), whereas those with higher education had a mean total ZBI score of 14.81 (SD = 10.25).

Similarly, when examining monthly income, caregivers earning less than 5000 SR had a mean burden score of 13.39 (SD = 8.99), whereas those earning more than 20000 SR had the highest mean total ZBI score (M = 15.50, SD = 11.53).

In addition, mean total ZBI scores also differed by living arrangement. Caregivers who did not live with their patients had lower mean total ZBI scores (M = 9.73, SD = 7.81) than those who did (M = 15.61, SD = 10.19).

Descriptive statistics for the Zarit Burden Interview (ZBI) by patients’ socio-demographic characteristics were illustrated in Table 2. Regarding patient age, the largest age category was younger than 18 years (n = 28, 23.3%), followed by 18–24 years (n = 24, 20.0%). Caregiver burden varied across patient age groups, with the highest mean ZBI score observed among caregivers of patients aged 55–64 years (Mean = 16.00, SD = 10.96). The relationship between caregiver and patient was associated with differences in caregiver burden. Spouse caregivers had the highest mean ZBI total score (M = 17.50, SD = 12.04). This was followed by sons or daughters (M = 17.04, SD = 9.93) and parents (M = 16.08, SD = 10.02). Siblings had a lower mean ZBI score (M = 12.20, SD = 9.97). Caregivers categorized as “others,” which include aunt, uncle, and neighbors, had the lowest mean total ZBI score (M = 9.21, SD = 6.81).

**Table 2:** Descriptive statistics of Zarit Burden Interview scores (ZBI) by patient socio-demographic characteristics.

		N	Mean	Standard Deviation
Patinet’s age	Less than 18	28	14.39	8.68
	18-24	24	12.21	10.35
	25-34	18	14.22	10.38
	35-44	15	14.33	11.86
	45-54	11	12.27	9.39
	55-64	10	16.00	10.96
	>65	14	13.86	9.53
Relationship to caregiver	Parent	25	16.08	10.02
	Sibling	45	12.20	9.97
	Son/Daughter	27	17.04	9.93
	Spouse	4	17.50	12.04
	Others	19	9.21	6.81
Type of mental illness	Schizophrenia	24	11.83	10.51
	ADHD	5	15.00	9.46
	Autism	22	14.23	9.45
	Bipolar disorder	18	16.00	9.94
	Depressive disorder	29	13.03	10.19
	OCD	12	12.17	7.18
	Personality disorder	10	17.20	12.08
Duration of mental illness	1 year or less	9	12.00	11.62
	1–5 years	36	13.17	9.70
	10–15	15	12.47	7.84
	5–10 years	26	14.42	10.28
	More than 15	34	15.06	10.43
Severity of mental illness	Low	20	10.15	6.67
	Moderate	60	12.32	8.83
	Severe	30	15.87	9.84
	Very severe	10	23.80	14.20

The type of mental illness was associated with differences in caregiver burden. Caregivers of patients with personality disorders had a mean total ZBI score of 17.20 (SD = 12.08), whereas caregivers of patients with schizophrenia (M = 11.83, SD = 10.51), OCD (M = 12.17, SD = 7.18), and autism (M = 14.23, SD = 9.45) had lower mean total ZBI scores.

In addition, the duration of mental illness was also associated with caregiver burden. Caregivers of patients with more than 15 years of illness had a higher mean total ZBI score (M = 15.06, SD = 10.43) than those caring for patients with shorter illness duration.

### Correlations between caregiver burden and psychological variables

Our results demonstrated a statistically significant negative correlation between caregivers’ perceived burden and the physical (r = −0.428, p ≤ 0.001) and psychological (r = −0.227, p = 0.013) domains. A statistically significant positive correlation was observed between caregiver burden and depression (r = 0.397, p < 0.001) and anxiety symptoms (r = 0.538, p < 0.001). Other correlations were not statistically significant. See Table 3.

**Table 3:** Correlation analysis of Quality of Life Domains, PHQ-9, and GAD-7 Scores with Zarit Burden Interview Scores using Karl Pearson’s Coefficient of Correlation.

Variable (with ZBI scores)	Pearson Coefficient (r)	p-value
**Physical**	−.428	<.001**
**Psychological**	−.227	.013*
**Environmental**	−.158	.085
**Social**	−.175	.056
**GAD-7**	.538	<.001**
**PHQ-9**	.397	<.001**

* The correlation was significant at the 0.05 level (2-tailed). ** The correlation was significant at the 0.01 level (2-tailed).

### Group comparisons of caregiver burden

Table 4 presents the results of the independent samples t-tests. Notably, a statistically significant difference in burden scores was observed between male and female caregivers (t = −2.073, p = 0.04). Females had higher mean burden scores (15.74 ± 9.76) than males (12.05 ± 9.72), with a mean difference of −3.689 (95% CI: −7.214 to −0.164).

**Table 4:** Results of t-test with caregivers burden scores (ZBI) (N = 120).

Variable		N	Mean (SD)	Mean Difference	t-Value	p-Value	95% CI of the Difference
**Gender**	Males	63	12.05 (9.72)	−3.689	−2.073	0.04	−7.214– −0.164
	Female	57	15.74 (9.76)				
**Living with the patient**	Yes	83	15.61 (10.19)	5.885	3.125	0.002*	2.156–9.614
	No	37	9.73 (7.81)				

* The correlation was significant at the 0.05 level (2-tailed).

A statistically significant difference in burden scores was also observed between caregivers living with the patient and those who did not (t = 3.125, p = 0.002). Caregivers living with the patient had higher mean burden scores, with a mean difference of 5.885 (95% CI: 2.156 to 9.614).

One-way ANOVA analyses found that the severity of mental illness was significantly associated with caregiver burden (F = 5.863, p = 0.001). Additionally, the relationship with the caregiver was also significantly associated with caregiver burden (F = 2.663, p = 0.036). In contrast, monthly income was not significantly associated with caregiver burden (F = 0.443, p = 0.723). See Table 5.

**Table 5:** Comparison of Mean Zarit Burden Interview (ZBI) Scores using One-Way ANOVA (N = 120).

Variable		N	Mean (SD)	F-Value	p-Value
**Severity of Mental Illness**	Low	20	10.15 (6.67)	5.863	.001
	Moderate	60	12.32 (8.83)		
	Severe	30	15.87 (9.84)		
	Very severe	10	23.80 (14.20)		
**Monthly income**	Less than 5000	33	13.39 (8.99)	.443	.723
	5000-10000	19	13.37 (8.89)		
	10000-20000	36	12.89 (9.76)		
	More than 20000	32	15.50 (11.53)		
**Relationship with the caregiver**	Parent	25	16.08 (10.02)	2.663	.036
	Sibling	45	12.20 (9.97)		
	Son/Daughter	27	17.04 (9.93)		
	Spouse	4	17.50 (12.04)		
	Others	19	9.21 (6.81)		

### Multivariable linear regression analysis of caregiver burden

To identify independent predictors of caregiver burden, an explorative multivariable linear regression analysis was performed with the mean Zarit Burden Interview (ZBI) score as the dependent variable (Table 6). All variables identified as clinically, theoretically and practicably relevant from the literature and the preliminary univariable analyses were initially considered. Predictor selection was guided through iterative model building and educated literature review while monitoring multicollinearity diagnostics and overall model performance. Variables demonstrating minimal contribution to the model (typically p > .450) were sequentially removed to obtain a more parsimonious and stable model, particularly in light of the relatively modest sample size (N = 120). Collinearity diagnostics indicated no evidence of problematic multicollinearity except the subscales of the Quality of life when tested simultaneously in the same iterative model, and the final model satisfied the assumptions required for linear regression analysis. The final regression model was statistically significant, f(6,113) = 11.12, p < .001, explaining 37.1% of the variance in caregiver burden scores (R² = .371; adjusted R² = .338). This indicates that the predictors retained in the final model collectively accounted for approximately one-third of the variability in perceived caregiver burden. After adjustment for the other variables in the model, caregivers’ anxiety symptoms emerged as the strongest independent predictor of burden. Specifically, each one-point increase in the GAD-7 anxiety score was associated with an average increase of 0.88 points in the ZBI burden score (B = 0.878, 95% CI [0.521, 1.236], p < .001). This finding suggests that higher levels of perceived anxiety are independently associated with greater caregiving burden.

**Table 6:** Multivariable linear regression analysis of caregivers mean perceived Burden (ZBI) Score N = 148 data records.

	Unstandardized Beta coefficient	95% CI for Beta coefficient		
		Lower	Upper	p-value
(Constant)	−0.444	−8.791	7.903	0.916
Sex = Male	0.750	−2.593	4.093	0.657
Age (years)	0.049	−0.063	0.161	0.386
Living with the patient = Yes	2.905	−0.474	6.283	0.091
Severity of mental illness = severe	2.652	0.810	4.494	0.005
Mean perceived Generalized Anxiety (GAD7) score	0.878	0.521	1.236	<0.001
Mean perceived Quality Of Life (QoL) score	0.004	−0.093	0.101	0.934

Dependent Variable: Caregiver mean perceived Zarit Burden Interview score. Model overall significance: f(6,113)=11.12, p-value<0.001. Model R-squared = 0.371, adjusted R-squared = 0.338.

Perceived severity of the patient’s mental illness was also independently associated with caregiver burden. Caregivers who perceived the patient’s illness as severe reported burden scores that were, on average, 2.65 points higher than the reference category (low to mild) after controlling for other factors (B = 2.652, 95% CI [0.810, 4.494], p = .005). This finding indicates that greater perceived illness severity is associated with increased caregiver strain. Living with the patient demonstrated a positive but non-significant trend toward higher burden scores (B = 2.905, 95% CI [−0.474, 6.283], p = .091). Although the association did not reach conventional statistical significance, the direction and magnitude of the coefficient suggest a potentially meaningful relationship that may warrant further investigation in larger studies.

In contrast, caregiver sex (B = 0.750, p = .657), age (B = 0.049, p = .386), and overall quality of life score (B = 0.004, p = .934) were not independently associated with caregiver burden after adjustment for the other predictors. Although some of these variables showed associations in the univariable analyses, their effects were attenuated in the multivariable model, suggesting that their relationships with burden may be explained by shared variance with anxiety levels and perceived illness severity.

### Multivariable linear regression analysis of caregivers’ quality of life

To further examine factors independently associated with caregivers’ quality of life (QoL), a multivariable linear regression analysis was performed using the overall QoL score as the dependent variable (Table 7). Consistent with the caregiver burden model, all clinically and theoretically relevant variables identified from the literature and preliminary analyses were initially evaluated. Model development followed an iterative approach, with predictor retention guided by statistical significance, collinearity diagnostics, model fit indices, and clinical relevance. Variables demonstrating limited explanatory value were removed to achieve a more parsimonious model while minimizing the risk of overfitting given the available sample size. The final model was statistically significant, f(8,111) = 14.04, p < .001, and explained approximately 50.3% of the variance in caregivers’ quality of life scores (R² = .503; adjusted R² = .467). This level of explained variance suggests that the model accounted for a substantial proportion of the variability in perceived quality of life among caregivers.

**Table 7:** Multivariable linear regression analysis of caregivers mean perceived Quality of Life (QoL) score.

	Unstandardized Beta coefficient	95% CI for Beta coefficient		
		Lower	Upper	p-value
(Constant)	59.470	47.187	71.754	<0.001
Sex = Male	3.106	−2.814	9.025	0.301
Age (years)	0.173	−0.025	0.372	0.086
Duration of mental illness	1.310	−0.755	3.374	0.211
Living with the patient = Yes	7.187	1.226	13.148	0.019
Severity of mental illness	−1.674	−5.187	1.839	0.347
Mean perceived Generalized Anxiety (GAD7) score	−0.806	−1.607	−0.004	0.049
Mean perceived Zarit Burden Interview score	0.026	−0.305	0.356	0.877
Mean perceived Depression (PHQ9) score	−1.417	−2.031	−0.804	<0.001

Dependent Variable: Caregiver mean perceived Zarit Burden Interview score. Model overall significance: f(8,111)=14.04, p-value<0.001. Model R-squared = 0.503, adjusted R-squared = 0.467.

After adjustment for all other predictors, depressive symptoms emerged as the strongest independent correlate of quality of life. Higher PHQ-9 depression scores were significantly associated with lower QoL scores (B = −1.417, 95% CI [−2.031, −0.804], p < .001). Specifically, each one-point increase in depression score was associated with an average reduction of approximately 1.4 points in the overall QoL score.

Anxiety symptoms also demonstrated an independent inverse association with quality of life. Higher GAD-7 scores were associated with lower QoL scores (B = −0.806, 95% CI [−1.607, −0.004], p = .049), indicating that caregivers experiencing greater anxiety tended to report poorer quality of life, even after controlling for depressive symptoms and other relevant factors. Living with the patient was independently associated with higher QoL scores (B = 7.187, 95% CI [1.226, 13.148], p = .019). Caregivers residing with the patient reported QoL scores that were, on average, approximately seven points higher than those not living with the patient after adjustment for the other variables in the model. While this finding may appear counter-intuitive given the increased caregiving demands associated with co-residence, it may reflect stronger family support structures, enhanced caregiver adaptation, greater involvement in patient care, or cultural factors that were not directly measured in the present study.

Several variables were not independently associated with quality of life after adjustment. These included caregiver sex (B = 3.106, p = .301), duration of mental illness (B = 1.310, p = .211), perceived severity of mental illness (B = −1.674, p = .347), and caregiver burden score (B = 0.026, p = .877). Age demonstrated a borderline association with QoL (B = 0.173, 95% CI [−0.025, 0.372], p = .086), suggesting a possible trend toward better quality of life among older caregivers, although this relationship did not reach statistical significance. Too, the other study measured predictor independent relevant factors did not correlate significantly with the caregivers' perceived QoL score when tested in iterative models conducted by the researchers as such were dismissed from the final model.

Of particular interest, caregiver burden was no longer significantly associated with quality of life after controlling for anxiety and depressive symptoms. This finding suggests that the previously observed bivariate relationship between burden and quality of life may be largely explained by underlying psychological distress, particularly depression and anxiety.

## 4. Discussion

Our study found a moderate, statistically significant negative correlation between caregivers’ perceived burden and QoL, primarily affecting the physical and psychological domains. This finding suggests an inverse association between caregiver burden and QoL, whereby higher burden scores were associated with lower QoL scores. Additionally, we found a significant positive correlation among caregiver burden, depression, and generalized anxiety scores. This finding suggests that higher caregiver burden is associated with greater psychological distress. Our results showed that caregivers living with patients reported significantly higher levels of burden, underscoring the added stress of constant proximity to patients. Our study also found a relationship between caregiver burden and the severity of patients’ mental illness, indicating that the severity of mental illness was significantly associated with caregiver burden.

The profound psychological distress faced by caregivers of individuals with SPMI is well-documented in the literature [1,8–10]. Specifically, Fekadu et al. [12] reported that caregivers of individuals with severe mental illness experience substantial psychological, social, and economic burdens, including emotional distress, reduced quality of life, social isolation, and financial strain. These multidimensional impacts may explain the observed associations between caregiver burden, poorer QoL, and higher levels of anxiety and depression in our sample. Our study corroborated these findings, revealing a significant negative correlation between caregiver burden and psychological domain scores on the WHOQOL-BREF, as well as positive correlations with anxiety and depression scores. This consistent association underscores the significant psychological challenges caregivers face.

The findings of our study showed that female caregivers of patients with SPMI experienced a higher burden, which is consistent with previous research on related populations. Hsiao et al. [21] reported that female family caregivers of individuals with schizophrenia experienced a more significant burden than did male caregivers. Similarly, another study by Van Wijngaarden et al. [22] demonstrated that among caregivers of individuals with depression, those facing the most substantial burden were predominantly female. In addition, Al‑Farsi et al. [23] observed that among parents caring for children with autism spectrum disorder, mothers suffered more psychological distress than fathers. These studies underscore a consistent trend across psychiatric conditions, highlighting the importance of considering sex-specific challenges when designing supportive interventions for caregivers. This finding may reflect the greater caregiving responsibilities traditionally assumed by women in many cultures, including Saudi Arabia, where women are often the primary providers of emotional and practical care within the family. Balancing caregiving responsibilities with household, parenting, and occupational demands may contribute to increased caregiver burden among female caregivers.

Consistent with the findings of Souza et al. [11], our study found that living with a patient with SPMI was significantly associated with higher caregiver burden. One possible explanation is that co-residing caregivers may face greater caregiving demands and more frequent exposure to care recipients’ difficulties, which could contribute to higher perceived burden. Furthermore, co-residing caregivers may have fewer opportunities for respite and recovery from caregiving responsibilities, resulting in sustained psychological and emotional strain. Continuous exposure to patients’ symptoms and functional difficulties may further intensify perceptions of burden. Interestingly, our multivariable analysis also showed that caregivers who lived with the patient reported higher quality of life after adjustment for other factors. Although this finding may appear counterintuitive, caregiver burden and quality of life are related but distinct constructs and may be influenced by different underlying factors. In the Saudi cultural context, where caregiving responsibilities are often shared within extended families and supported by strong familial and religious values, living with the patient may foster a greater sense of purpose, emotional connectedness, and access to family support, which could partially offset the negative impact of caregiving on overall quality of life. It is also possible that unmeasured factors not captured in the present study contributed to this association. Therefore, this finding should be interpreted cautiously and warrants confirmation in larger, diagnosis-specific longitudinal studies.

Our findings regarding the impact of socioeconomic status on caregiver burden differ from those of Siddiqui and Khalid [13]. While they found a significantly higher caregiver burden among family members with lower monthly income, our study did not find a significant association between income level and caregiver burden. This discrepancy indicates that other factors, such as cultural differences, availability of community resources, and differences in healthcare systems, could affect the relationship between financial strain and caregiver burden. The availability of free healthcare in Saudi Arabia and the presence of government social assistance programs for patients with chronic illnesses, such as the Comprehensive Rehabilitation Program, may partially account for the lack of a significant association between income level and caregiver burden observed in this study.

Our study identified a significant association between the perceived severity of mental illness and caregiver burden. Similarly, a study conducted in the USA found a positive correlation between increased psychiatric symptoms and a higher subjective caregiver burden [24]. It is important to note that, in our study, the severity of mental illness was assessed through self-reported assessments of the perceptions of severity, reflecting the caregivers’ perceptions of the mental health challenges faced by the patient rather than a more objective clinical assessment. More severe psychiatric symptoms often require increased supervision, emotional support, assistance with daily functioning, and management of behavioral disturbances, all of which may increase caregiving demands and contribute to higher levels of perceived burden.

Researchers found that caregivers for close family members reported the highest burden scores [25]. Similarly, our study found a significant association between caregiver-patient relationships and burden scores: those caring for parents, children, or spouses reported higher burden scores than those caring for more distant relatives or non-family caregivers. The emotional and practical demands associated with close familial relationships may contribute to these findings and suggest that this group could particularly benefit from targeted support interventions.

Furthermore, multivariable linear regression analysis demonstrated that caregivers’ anxiety symptoms and perceived severity of the patient’s mental illness were independent predictors of caregiver burden after adjustment for demographic and caregiving-related factors. Anxiety emerged as the strongest predictor, indicating that psychological distress may play a central role in the caregiving experience. This finding is consistent with previous studies reporting that caregivers experiencing higher levels of anxiety and psychological distress tend to report greater caregiving burden [13,21,25]. Similarly, caregivers who perceived the patient’s illness as more severe reported significantly higher burden scores. Comparable findings have been reported by Greenberg et al. [24], who observed that greater psychiatric symptom severity was associated with increased subjective caregiver burden. These findings suggest that interventions aimed at reducing caregiver anxiety and supporting families caring for individuals with more severe mental illness may help alleviate caregiver burden.

The findings should be interpreted cautiously, given that the achieved sample size was smaller than the initial target sample size, which may have reduced statistical power and limited the detection of smaller associations. Although the study included caregivers of individuals with diverse psychiatric and neurodevelopmental disorders, no statistically significant differences in caregiver burden were observed across diagnostic groups. However, these findings should not be interpreted as evidence that caregiver experiences are equivalent across diagnoses. Rather, caregiving burden is likely influenced by multiple factors, including symptom severity, functional impairment, and caregiving responsibilities. Given the relatively small sample sizes within individual diagnostic groups, the study may have been under-powered to detect clinically meaningful differences.

## 5. Limitations

This study has several limitations. First, convenience sampling may have introduced selection bias; future research should use random sampling to enhance representativeness. Second, recruiting participants from a single tertiary care center may limit the generalizability of the findings to other regions or healthcare settings in Saudi Arabia. Large-scale national studies are needed for a more comprehensive understanding. Third, the cross-sectional design restricts causal inference. While significant associations were found, the direction of these relationships remains unclear. The sample size may have limited the detection of smaller associations. Furthermore, the final achieved sample size was lower than the initially calculated target sample size because of recruitment challenges encountered during the study period. Additionally, the study included caregivers of individuals with diverse psychiatric and neurodevelopmental disorders, including schizophrenia, bipolar disorder, autism spectrum disorder, and attention-deficit/hyperactivity disorder. Although no significant differences in caregiver burden or quality of life were observed across diagnostic categories in the present sample, the relatively small number of participants within individual diagnostic groups may have limited statistical power to detect diagnosis-specific effects. Therefore, the clinical heterogeneity of the sample should be considered when interpreting the findings. Future studies with larger, multi-center samples and longitudinal designs will offer a more complete understanding of caregiver experiences and the progression of burden over time.

## 6. Conclusions

This is the first study in Saudi Arabia to examine depression and anxiety in caregivers of individuals with severe mental illnesses and their association with caregiver burden and quality of life. Our findings emphasize the influence of caregiver psychological distress, particularly anxiety and depression, as well as co-residence and perceived illness severity, on caregiver burden and quality of life. Although no statistically significant differences were observed across diagnostic groups, these findings should be interpreted cautiously because the study was not powered to detect potentially meaningful differences between specific diagnostic categories. Future studies with larger, diagnosis-specific samples are needed to better understand potential differences in caregiver experiences across psychiatric conditions. These results highlight the need for caregiver-focused screening for anxiety, depression, and burden, as well as psychosocial support in mental health services. Strengthening support mechanisms may improve treatment adherence, reduce caregiver strain, enhance patient outcomes, and inform policies aimed at caregiver well-being.

### Informed consent statement

Written informed consent has been obtained from the patient(s) to publish this paper.
